# Cyclic AMP signaling in *Dictyostelium* promotes the translocation of the copine family of calcium-binding proteins to the plasma membrane

**DOI:** 10.1186/s12860-018-0160-5

**Published:** 2018-07-16

**Authors:** April N. Ilacqua, Janet E. Price, Bria N. Graham, Matthew J. Buccilli, Dexter R. McKellar, Cynthia K. Damer

**Affiliations:** Biology Department, Central Michigan University, Mount Pleasant, MI 48859 USA

**Keywords:** Copine, *Dictyostelium*, Calcium, Phospholipid, Membrane, cAMP

## Abstract

**Background:**

Copines are calcium-dependent phospholipid-binding proteins found in many eukaryotic organisms and are thought to be involved in signaling pathways that regulate a wide variety of cellular processes. Copines are characterized by having two C2 domains at the N-terminus accompanied by an A domain at the C-terminus. Six copine genes have been identified in the *Dictyostelium* genome, *cpnA – cpnF*.

**Results:**

Independent cell lines expressing CpnA, CpnB, CpnC, CpnE, or CpnF tagged with green fluorescent protein (GFP) were created as tools to study copine protein membrane-binding and localization. In general, the GFP-tagged copine proteins appeared to localize to the cytoplasm in live cells. GFP-tagged CpnB, CpnC, and CpnF were also found in the nucleus. When cells were fixed or when live cells were treated with calcium ionophore, the GFP-tagged copine proteins were found associated with the plasma membrane and vesicular organelles. When starved *Dictyostelium* cells were stimulated with cAMP, which causes a transitory increase in calcium concentration, all of the copines translocated to the plasma membrane, but with varying magnitudes and on and off times, suggesting each of the copines has distinct calcium-sensitivities and/or membrane-binding properties. In vitro membrane binding assays showed that all of the GFP-tagged copines pelleted with cellular membranes in the presence of calcium; yet, each copine displayed distinct calcium-independent membrane-binding in the absence of calcium. A lipid overlay assay with purified GFP-tagged copine proteins was used to screen for specific phospholipid-binding targets. Similar to other proteins that contain C2 domains, GFP-tagged copines bound to a variety of acidic phospholipids. CpnA, CpnB, and CpnE bound strongly to PS, PI(4)P, and PI(4,5)P_2_, while CpnC and CpnF bound strongly to PI(4)P.

**Conclusions:**

Our studies show that the *Dictyostelium* copines are soluble cytoplasmic and nuclear proteins that have the ability to bind intracellular membranes. Moreover, copines display different membrane-binding properties suggesting they play distinct roles in the cell. The transient translocation of copines to the plasma membrane in response to cAMP suggests copines may play a specific role in chemotaxis signaling.

**Electronic supplementary material:**

The online version of this article (10.1186/s12860-018-0160-5) contains supplementary material, which is available to authorized users.

## Background

Copines are a family of soluble calcium-dependent membrane-binding proteins [1]. Multiple copine homologs are found in a variety of organisms such as *Paramecium tetraurelia*, *Arabidopsis thaliana*, *Caenorhabditis elegans*, *Dictyostelium discoideum,* mice, and humans [1, 2]. The conservation of copine proteins in single-celled organisms to more complex eukaryotes, suggests copines play fundamental roles in eukaryotic cell processes. Copines are characterized by having two C2 domains located in the N-terminal half of the protein and an A-domain located in the C-terminal half [1].

C2 domains are protein motifs that confer calcium-dependent membrane-binding. Several studies have shown that copines, like other C2 domain containing proteins, are able to bind membranes in a calcium-dependent manner [1–6]. Many proteins that contain a single C2 domain are involved in signaling pathways and regulate the modification of lipids or protein phosphorylation. Most proteins containing multiple C2 domains regulate vesicle transport [7, 8]. The presence of C2 domains suggests copines play a role in cell signaling pathways and/or membrane trafficking. The A domain in copines is similar to the von Willebrand A (VWA) domain found in a number of extracellular matrix proteins and integrins. VWA domains are thought to function as protein-binding motifs facilitating protein-protein interactions and more specifically, multiprotein complexes [9]. Using yeast two-hybrid screening and pull-down experiments, Tomsig et al. [10] showed that the A domain of several human copines bind to many different intracellular proteins, which suggests copines have roles in a wide variety of cellular processes.

We are using the simple eukaryote *Dictyostelium discoideum* to study copine function. *Dictyostelium* lives as unicellular amoeba in the presence of a nutrition source, but develops into multicellular fruiting bodies when starved. In starvation conditions, single-celled amoebae aggregate together via cyclic adenosine monophosphate (cAMP) signaling and undergo morphogenesis into multicellular motile slugs that culminate into fruiting bodies during a 24-h life cycle. This developmental process can be used to study signaling pathways necessary for cellular processes such as chemotaxis, differentiation, and cell death [11].

The *Dictyostelium* genome contains six copine genes (*cpnA-cpnF*). The six copine proteins contain the characteristic two C2 domains followed by an A domain and exhibit 28–60% amino acid identity [2]. Expression patterns of the copines throughout development is different for each of the copines, suggesting they have distinct functions [12]. Vegetative cells lacking the *cpnA* gene display defects in cytokinesis and contractile vacuole function [12]. Developing *cpnA−* cells are delayed in aggregation, form larger than normal slugs, exhibit defects in slug thermotaxis and phototaxis, and fail to culminate into normal fruiting bodies [12–14]. GFP-tagged Copine A (GFP-CpnA) was shown to bind to membranes in a calcium-dependent manner and transiently translocate from the cytoplasm to the plasma membrane and intracellular vesicles in starved cells. Experiments with fixed cells showed that GFP-CpnA specifically associated with the plasma membrane, contractile vacuoles, phagosomes, and organelles of the endolysosomal pathway [2]. These data suggest that CpnA may bind membranes in response to signaling pathways that increase intracellular calcium concentrations and play a role in a variety of cellular processes that involve membrane trafficking and/or membrane remodeling.

To further our studies on CpnA and investigate whether the other copines have distinct or overlapping functions, we have expressed GFP-tagged versions of four additional copine proteins in *Dictyostelium discoideum*. We used these cells to characterize the calcium-dependent membrane-binding properties, lipid-binding specificities, and intracellular localization of each of the copines. In addition, we tested the hypothesis that copines translocate from the cytosol to membranes in response to a rise in intracellular calcium triggered by signaling molecules.

## Methods

### Dictyostelium cell culture

*Dictyostelium* cells were grown in HL-5 axenic media (0.75% proteose peptone #2, 0.75% proteose peptone #3, 0.5% yeast extract, 1% glucose, 2.5 mM Na_2_HPO_4_, and 8.8 mM KH_2_PO_4_, pH 6.5) supplemented with penicillin-streptomycin (60 U/mL) at 20 °C on either plastic dishes or in suspension shaking at 180 rpm. Plasmid transformed cells were cultured in media containing the antibiotic, G418 (9 μg/mL).

### Creation and sequencing of copine cDNA clones

Full-length (*cpnA*, *cpnB*, *cpnD*, *cpnE*) and partial (*cpnF*) cDNA clones were obtained from the Tsukuba *Dictyostelium discoideum* cDNA project based in Japan [15]: SLI395 for *cpnA*, CFH205 for *cpnB,* VFA828 for *cpnD*, VFK812 for *cpnE*, and VFL565 for *cpnF*. To obtain a cDNA clone for *cpnC*, RT-PCR was used to create cDNA from RNA isolated from the NC4A2 [16] axenic wild-type *Dictyostelium* strain. Because the *cpnD* cDNA clone obtained from the Japanese cDNA project contained a base deletion compared to the AX4 genomic sequence on dictyBase [17, 18], RT-PCR was also used to create *cpnD* cDNA clones from both NC4A2 and AX4 axenic wild-type *Dictyostelium* strains. However, the *cpnD* cDNAs obtained from RT-PCR contained differences and deletions in the same region. Therefore, we did not include *cpnD* in this study. All cDNAs were amplified by PCR with primers designed from the copine gene sequences on dictyBase [17, 18] that added either *KpnI* or *SacI* restriction sites to the ends of the clones. The downstream primers with *KpnI* sites did not contain the stop codon. Extended downstream primers were used to add the missing 29 bases to the 3′ end of the *cpnF* cDNA clone. PCR fragments were cloned into the pCR2.1-TOPO vector and sequenced. Sequences were analyzed using Sequencher 4.9 (Gene Codes Corporation) and compared to the copine gene sequences on dictyBase [17, 18]. The cDNA clone of *cpnB* used in this study had a single base difference (base 1392 A to G) that did not change the amino acid sequence from the sequence we reported earlier [2] (GenBank Accession#AY593970). The *cpnC* cDNA made from RNA from NC4A2 cells was compared to the protein coding sequence derived from genomic DNA from AX4 cells on dictyBase (DDB0216239) and we found 2 base changes: 906 T to C and 1515 A to G. Neither base difference changed the amino acid sequence. The *cpnE* cDNA sequence was the same as the protein coding sequence derived from the AX4 genomic DNA sequence (DDB0216242). The *cpnF* cDNA sequence had one base different (298 C to A) from the protein coding sequence derived from AX4 genomic DNA (DDB0233133) that changes a proline to a threonine.

### Creation of GFP-tagged copine expression plasmids

The cDNA clones of *cpnA*, *cpnB*, *cpnC*, *cpnE*, and *cpnF*, were subcloned into the *SacI* site of the pTX-GFP plasmid [19] for the expression of copines with a GFP tag at the N-terminus and the *KpnI* site of the pTX-GFP plasmid for expression of copines with a GFP tag at the C-terminus. The cDNA of *cpnA* was also subcloned into the *KpnI* site of the pDXA-GST plasmid [20] for the expression of CpnA with a glutathione-S-transferase (GST) tag at the N-terminus. The plasmids were transformed into NC4A2 *Dictyostelium* cells by electroporation [21], and the cells were plated on Petri dishes. Transformed cells were selected with G418 (9 μg/mL). Expression of GFP-tagged copine proteins was verified by Western blot with an antibody to GFP.

### Microscopy imaging of fixed cells

Cells expressing GFP or a GFP-tagged copine were harvested from plates, counted with a hemocytometer, centrifuged at 1500 rpm at room temperature (RT) for 5 min, resuspended at 2 × 10^6^ cells/mL in KK2 buffer (16.2 mM KH_2_PO_4_, 4.0 mM K_2_HPO4), and placed on coverslips in a humidity chamber for 15 min. Cells were flattened with a thin sheet of agarose and then fixed in a 1% formaldehyde in methanol solution at − 20 °C for 10 min. Fixed cells were washed three times with PBS (137 mM NaCl, 2.7 mM KCl, 4.3 mM Na_2_HPO_4_, 1.47 mM KH_2_PO_4_, pH 7.4) for 5 min each, treated with 0.05% Triton X-100 in PBS for 2 min, stained with 4′,6-diamidine-2′-phenylindole dihydrochloride (DAPI) (0.1 μg/mL) for 10 min, washed three times with PBS for 5 min each, and mounted on slides. Images were obtained with a Nikon Eclipse Ti inverted laser scanning confocal microscope using a 60× oil objective. Images were processed in Adobe Photoshop and levels of brightness and contrast were adjusted.

### Microscopy imaging of calcium ionophore-stimulated live cells

Cells expressing GFP or a GFP-tagged copine were harvested from plates, counted with a hemocytometer, centrifuged at 1500 rpm for 5 min at RT, and resuspended at 1 × 10^6^ cells/mL in KK2 buffer. Cells were plated on glass bottom dishes either in the presence of 2 mM CaCl_2_ or 5 mM EGTA in KK2 buffer and allowed to sit for 15 min prior to ionophore treatment. Cells were globally stimulated with 10 μM calcium ionophore, A23187 (Sigma, C7522), in KK2 buffer supplemented with either 2 mM CaCl_2_, 5 mM CaCl_2_, or 5 mM EGTA. Images were obtained 20–300 s after treatment with the ionophore with a Nikon Eclipse Ti inverted laser scanning confocal microscope using a 60× oil objective. Images were processed in Adobe Photoshop and levels of brightness and contrast were adjusted.

### Microscopy imaging of cAMP-stimulated live cells

Cells were harvested from plates, counted with a hemocytometer, centrifuged at 1500 rpm for 5 min at RT, washed twice with KK2 buffer, and resuspended in KK2 buffer at 5 × 10^6^ cells/mL. The cells were starved for 1 h in shaking suspension at 150 rpm at 20 °C and subsequently pulsed with 50 nM cAMP every 6 min for 5 h with shaking at 150 rpm. Aggregate competent cells were plated on glass bottom dishes in KK2 buffer with 2 mM caffeine and 2 mM CaCl_2_. Cells were stimulated with cAMP solution (5 μM cAMP, 2 mM CaCl_2_, 2 mM caffeine) and imaged prior to stimulation and after stimulation every 3 s with time-lapse imaging. Images were obtained with an inverted Nikon Eclipse Ti laser scanning confocal microscope with a 60× oil objective. Images were processed in Adobe Photoshop and levels of brightness and contrast were adjusted. Time-lapse images were converted to avi movies at 2 frames per second using ImageJ. Images of cells (at least 6 and up to 21 for each copine) in which copine translocation in response to cAMP stimulation was observed were analyzed for when plasma membrane association was first and last observed. The membrane on and off times for each copine, regardless of the position of the GFP tag, were estimated and the average times calculated. Relative fluorescence intensities of the plasma membrane and cytosol were measured from images of single cells using ImageJ; four defined regions of interest in both the cytosol and membrane were gated in images of cells taken before and after cAMP stimulation and used to calculate a mean fluorescence intensity. Percent difference was calculated by dividing the difference between the mean fluorescence intensity of the plasma membrane and the cytosol by the average of the two. Mean percent difference was calculated from six individual cells for each copine.

### Glutathione agarose chromatography

Cells expressing GST-tagged CpnA were harvested from plates and counted with a hemocytometer. The cells (5 × 10^8^) were pelleted at 1500 rpm for 5 min at 4°C and the supernatant was discarded. Cells were resuspended in lysis buffer (PBS containing 5 mM EGTA, 1% Triton X-100, 1 mM dithiothreitol (DTT), and protease inhibitor cocktail (Thermo Scientific, 78,415) and incubated on ice for 30 min. Glutathione agarose beads (Sigma, G0924) were suspended in 5 mL of deionized water, placed on the rotator, and incubated at RT for 30 min. The beads were washed three times in PBS by pelleting at 3000 rpm for 5 min at 4°C. The beads were resuspended in 150 μl of PBS to make a 50% bead slurry. Lysed cells were spun in a microfuge at 14,000 rpm for 5 min at 4°C. The cell lysate supernatant was added to the bead slurry and placed on the rotator at RT for 30 min. The beads were washed by pelleting at 3000 rpm for 5 min at 4°C four times in PBS. The beads were resuspended in elution buffer (10 mM reduced glutathione in 50 mM Tris-HCl, pH 8.0) and placed on a rotator for 10 min. The beads were pelleted and the supernatant containing the eluted GST-CpnA was stored at − 20°C. The concentration of the GST-CpnA was determined using the BIO-RAD protein assay kit.

### Native membrane binding assay

Cells were harvested from plates, counted with a hemocytometer, and centrifuged at 1500 rpm for 5 min at 4 °C. The supernatant was discarded and cells (2 × 10^7^) were resuspended in 200 μl of homogenization buffer (50 mM HEPES, 150 mM KCl, pH 7.4) with protease inhibitor cocktail. Beads (Zymo Research, S6002–50) were added and the cells were disrupted with a FastPrep FP120 cell disruptor for four 15-s cycles. Cells were kept on ice for 2 min between cycles. Cell lysates were removed from the bashing beads and divided equally into two tubes. CaCl_2_ or EGTA was added to the lysates to final concentrations of 2 mM and 5 mM, respectively. Lysates were spun in a Beckman Coulter air-driven ultracentrifuge at 100,000 x g for 45 min. The pellets and supernatants were resuspended in sample buffer and analyzed by Western blot with an antibody to GFP. Bands on the Western blots were quantified by densitometry analysis with ImageJ. Each native membrane binding assay was performed at least three times, the average percent of copine protein found with the pelleted membranes was calculated. For cells expressing GFP-CpnF and CpnF-GFP, we also performed these assays, adding the EGTA or CaCl_2_ to the buffer before cell disruption, and adding 10 mM EGTA instead of 5 mM. Cells expressing GFP-CpnF were treated with 5 μM Latrunculin A (Enzo Life Sciences, BML-T119–100) or 0.02% DMSO (Sigma, D4540) for 30 min prior to cell disruption.

### Western blot analysis

Protein samples were subjected to sodium dodecyl sulfate polyacrylamide gel electrophoresis (SDS-PAGE) and then transferred to polyvinylidene difluoride (PVDF) membrane. The membrane was incubated with blocking buffer (5% dried milk in TBS-Tween (10 mM Tris-HCl, pH 8.0, 100 mM NaCl, 0.1% *v*/v Tween-20)) for 1 h at RT and then incubated with a mouse anti-GFP antibody (Santa Cruz Biotechnology, SC-9996) or a mouse anti-actin antibody (Santa Cruz Biotechnology, SC-47778) (1:2000) in blocking buffer for 12 h at 4 °C overnight, washed with TBS-T, and incubated with a goat anti-mouse secondary antibody conjugated to horseradish peroxidase (HRP) (Thermo Scientific, 31,430) (1:15,000) for 1 h at RT. The membrane was washed with TBS-Tween and incubated with GE Healthcare ECL Western blotting detection solution and imaged with a BIO-RAD ChemiDoc Touch imaging system. Cells expressing GST-CpnA were also used in the native membrane binding assay and the GST was detected with an HRP-conjugated anti-GST antibody (1:5000) (GE Healthcare Life Services, RPN 1236 V).

### Immunoprecipitation and lipid dot blot assay

Cells were harvested from plates, counted with a hemocytometer, and centrifuged at 1500 rpm for 5 min at RT. Cells (1 × 10^8^) were resuspended in 5 ml of 50 mM HEPES (pH 7.4), centrifuged at 1500 rpm for 5 min at RT, and then resuspended in 1.5 ml of lysis buffer (50 mM HEPES, pH 7.4, 100 mM NaCl, 2 mM EGTA, 10% w/v sucrose, 0.3% v/v Nonidet P-40, protease inhibitor cocktail), and placed on the rotator at RT for 10 min. To prepare magnetic Protein G Dynabeads (Novex, 100,031), the Dynabeads were vortexed and 50 μl transferred to a microcentrifuge tube; the magnetic strip was used to isolate the magnetic Dynabeads and the supernatant removed. The beads were washed three times with 50 μl of PBS, resuspended in 200 μl of PBS, and incubated for 10 min at RT with 2 μl of a rabbit polyclonal anti-GFP antibody (Abcam AB290). Cell lysates were centrifuged at 14,000 rpm for 10 min. The lysate supernatant was incubated with the antibody-prepared Dynabeads for 30 min at RT. The beads were washed three times with 50 mM HEPES (pH 7.4) using the magnetic strip and then resuspended in 90 μl of 0.1 M glycine (pH 2.5). The eluate was removed from the beads and transferred to a tube containing 10 μl of 1 M Tris HCl (pH 8.5). Lipid dot blots (Echelon, P6002) were incubated with blocking buffer (3% Bovine Serum Albumin (BSA) in TBS-T) for 1 h. The blots were then incubated with blocking buffer containing 2 mM CaCl_2_ and the immunoprecipitation eluate for 1 h at RT. The blots were washed three times with blocking buffer with 2 mM CaCl_2_ for 10 min each and then incubated with blocking buffer with 2 mM CaCl_2_ and a polyclonal anti-rabbit secondary antibody conjugated to HRP (1:15,000) (Sigma, A9169) for 2 h at RT. The blot was washed three times with blocking buffer with 2 mM CaCl_2_ for 5 min each at RT. The blots were incubated with GE Healthcare ECL Western blotting detection kit and imaged with a BIO-RAD ChemiDoc Touch imaging system for chemiluminescence. Purified GST-CpnA (0.5 μg/mL), instead of the eluate, was also used as a control in the lipid dot blot assay. An HRP-conjugated anti-GST antibody (1:5000) was used to detect the GST-CpnA on the blot. The dot blot assay for each protein was performed at least three times.

### Sequence analysis of the C2 domains of the Dictyostelium copines

The amino acids that make up the two C2 domains of the six *Dictyostelium* copines were identified using the NCBI Conserved Domain Search [22]. The two C2 domains and intervening sequences for all the copines were aligned and the percent amino acid identity calculated using Clustal Omega [23]. BoxShade was used to indicate conserved residues within the aligned sequences [24].

## Results

### Copines localize to the plasma membrane and intracellular membranes in methanol fixed cells

To investigate the intracellular location of copines in *Dictyostelium*, the cDNAs for *cpnA*, *cpnB*, *cpnC*, *cpnE*, and *cpnF* were subcloned into the pTX-GFP vector at the *SacI* site for the overexpression of copines tagged with GFP at the N-terminus (GFP-Cpn) and cloned into the *KpnI* site for the overexpression of copines tagged with GFP at the C-terminus (Cpn-GFP). The plasmids were transformed into *Dictyostelium* NC4A2 cells, a wild-type axenic strain*.* Expression of the GFP-tagged copines in *Dictyostelium* cells was verified by Western blot (Additional file 1). In a previous study, we showed that CpnA with GFP at the N-terminus (GFP-CpnA) primarily localized to the cytoplasm in live cells, but was found associated with membranes when fixed with formaldehyde in methanol. These membranes included the plasma membrane, and the membranes of contractile vacuoles, organelles of the endolysosomal pathway, and phagosomes [2].

**Fig. 1:**
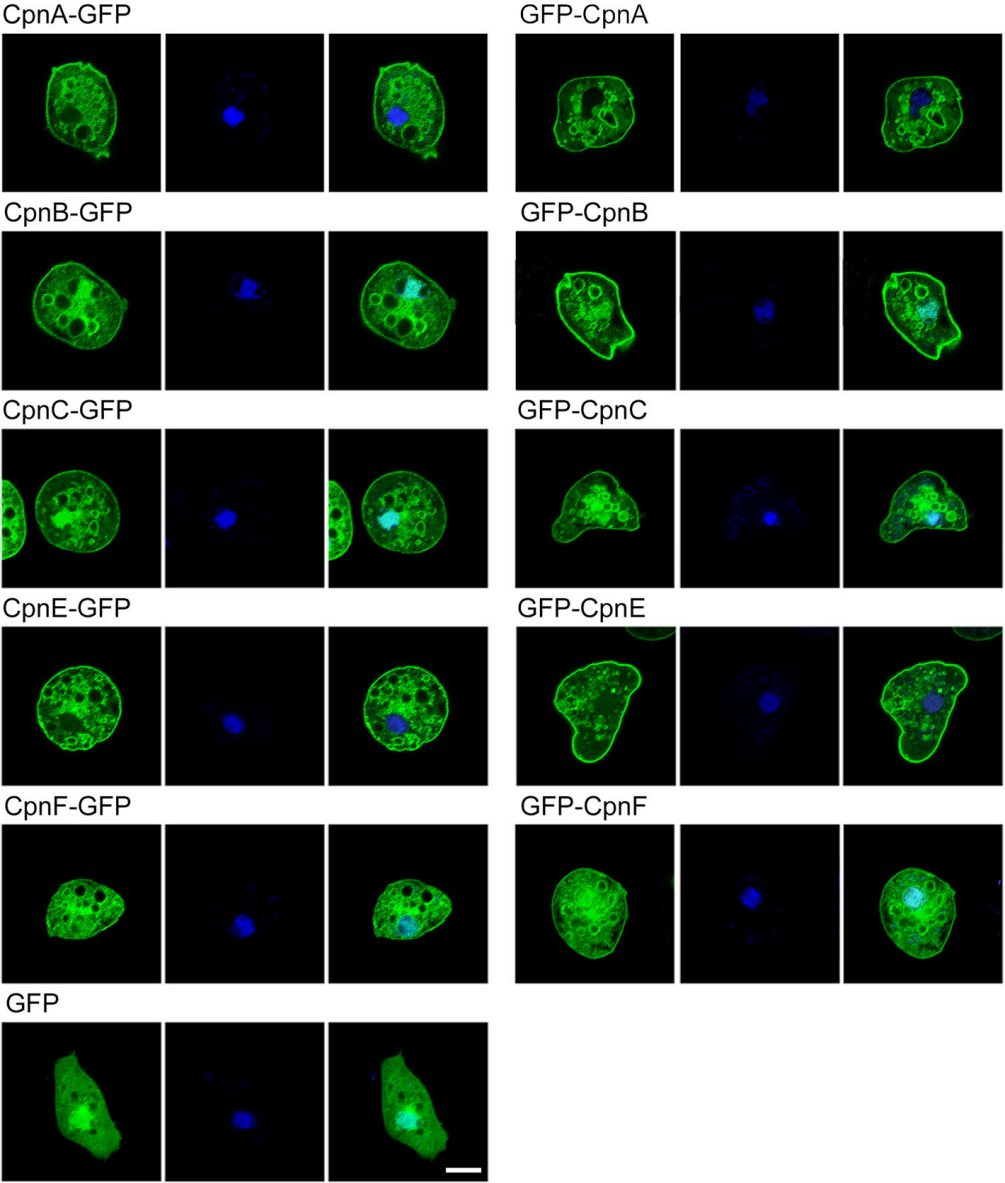
Localization of GFP-tagged copine proteins in fixed cells. Cells expressing GFP or a GFP-tagged copine were fixed in 1% formaldehyde in cold methanol and stained with 0.1 μg/ml DAPI. Three images of a representative cell from each cell line are shown. The first image shows the GFP fluorescence, the second shows the DAPI staining, and the third is a merged image showing both GFP and DAPI. Images were obtained with a Nikon Eclipse Ti inverted laser scanning confocal microscope using a 60× oil immersion objective. Scale bar = 5 μm

Cells expressing the GFP-tagged versions of copines were fixed with 1% formaldehyde in cold methanol, treated with DAPI to stain the nuclei, and then imaged by laser scanning confocal microscopy (Fig. 1). Representative images are shown in Fig. 1. All of the GFP-tagged copines were found associated with the plasma membrane and various intracellular vesicles and organelles. In addition, GFP-tagged versions of CpnB, CpnC, and CpnF were most often found in the nucleus, while CpnA and CpnE were never observed in the nucleus. The terminus at which the GFP was located did not appear to affect the localization patterns of the copines. As a control, we imaged cells expressing GFP, which was found in the cytoplasm and nucleus in fixed cells (Fig. 1).

### Copines translocate to membranes in live cells when treated with a calcium ionophore

**Fig. 2:**
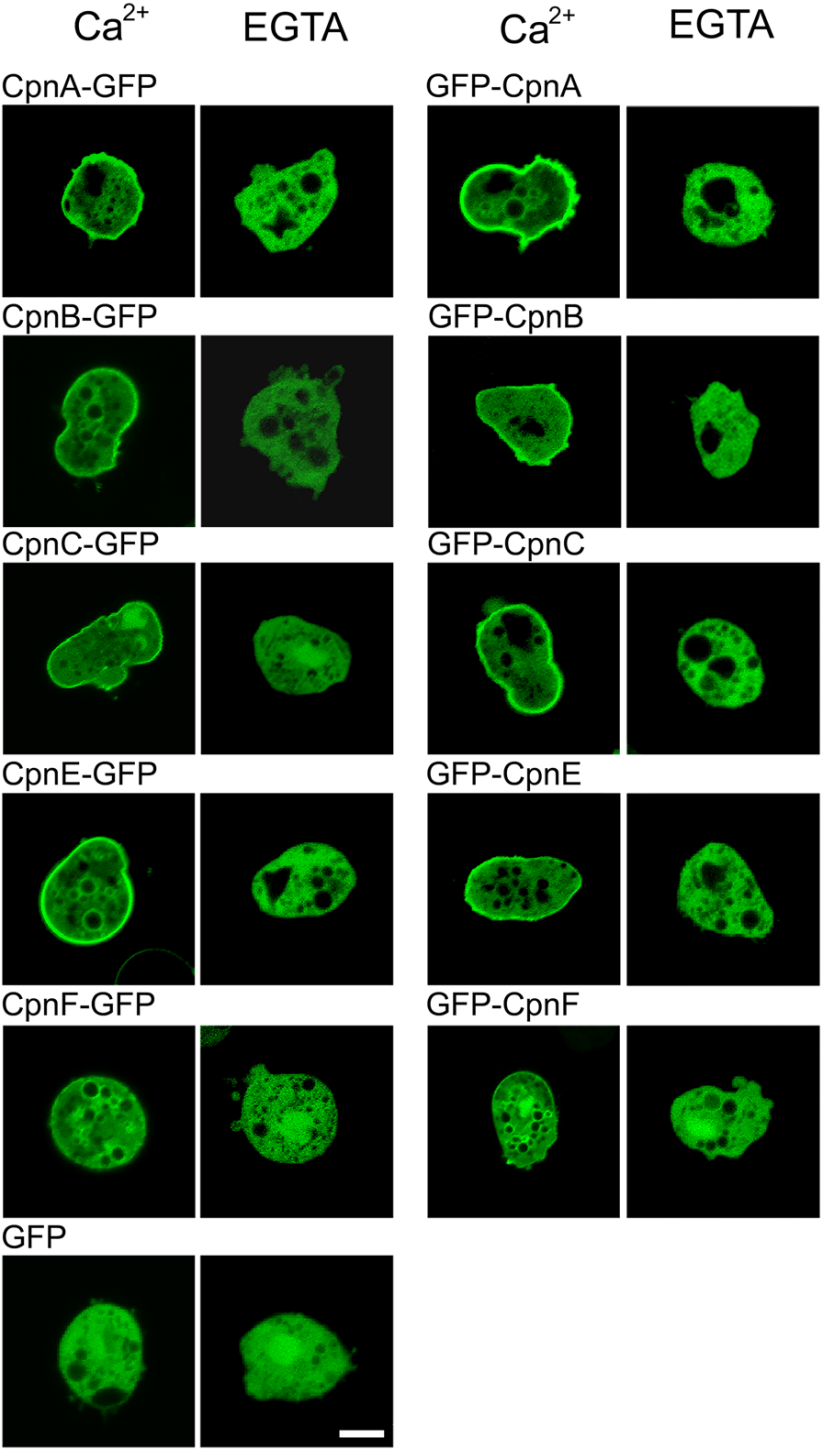
GFP-tagged copine proteins translocate to membranes in live cells treated with calcium ionophore. Cells expressing GFP or a GFP-tagged copine were treated with calcium ionophore in the presence of 2 mM CaCl_2_ or 5 mM CaCl_2_ for CpnF (Ca^2+^) or 5 mM EGTA (EGTA). Images were obtained with a Nikon Eclipse Ti inverted laser scanning confocal microscope using a 60× oil immersion objective. Representative images are shown for each copine cell line. Scale bar = 5 μm

In live vegetative cells, GFP-tagged versions of copines were found throughout the cytoplasm, and in the nucleus in the case of CpnB, CpnC, and CpnF. This result is similar to what we observed in our previous study with cells expressing GFP-CpnA [2], where GFP-CpnA appeared to be mostly cytoplasmic in live cells but associated with membranes in fixed cells. The difference in localization between live and fixed cells may be because the fixation process releases calcium from intracellular stores, and therefore reveals copine localization in a calcium-rich environment (Fig. 1). To investigate whether copines are able to translocate from the cytosol to membranes in response to a rise in intracellular calcium, vegetative cells were treated with a calcium ionophore to increase the concentration of intracellular calcium. Cells expressing GFP-tagged versions of copines were placed on glass bottom dishes in buffer containing calcium or EGTA and imaged using laser scanning confocal microscopy. All of the GFP-tagged versions of copines translocated to membranes when treated with calcium ionophore in the presence of calcium. The time it took for translocation after the ionophore was added ranged from 30 s to several minutes. This translocation did not occur when the cells were treated with the calcium ionophore in the presence of EGTA. Representative images are shown in Fig. 2. CpnF took the longest to translocate (7–8 min) and translocation required treatment with a higher concentration of calcium (5 mM instead of 2 mM). In addition, CpnF appeared to be more prominent on vesicular membranes than the plasma membrane compared to the other copines (Fig. 2). The terminus at which the GFP was located did not appear to affect the membrane localization. We did not observe a difference in nuclear localization in response to calcium ionophore treatment. These data suggest all of the copines are able to translocate from the cytosol to membranes in response to a rise in intracellular calcium concentration due to treatment with a calcium ionophore.

### Copines translocate to the plasma membrane in aggregate competent cells when stimulated with cAMP

**Fig. 3:**
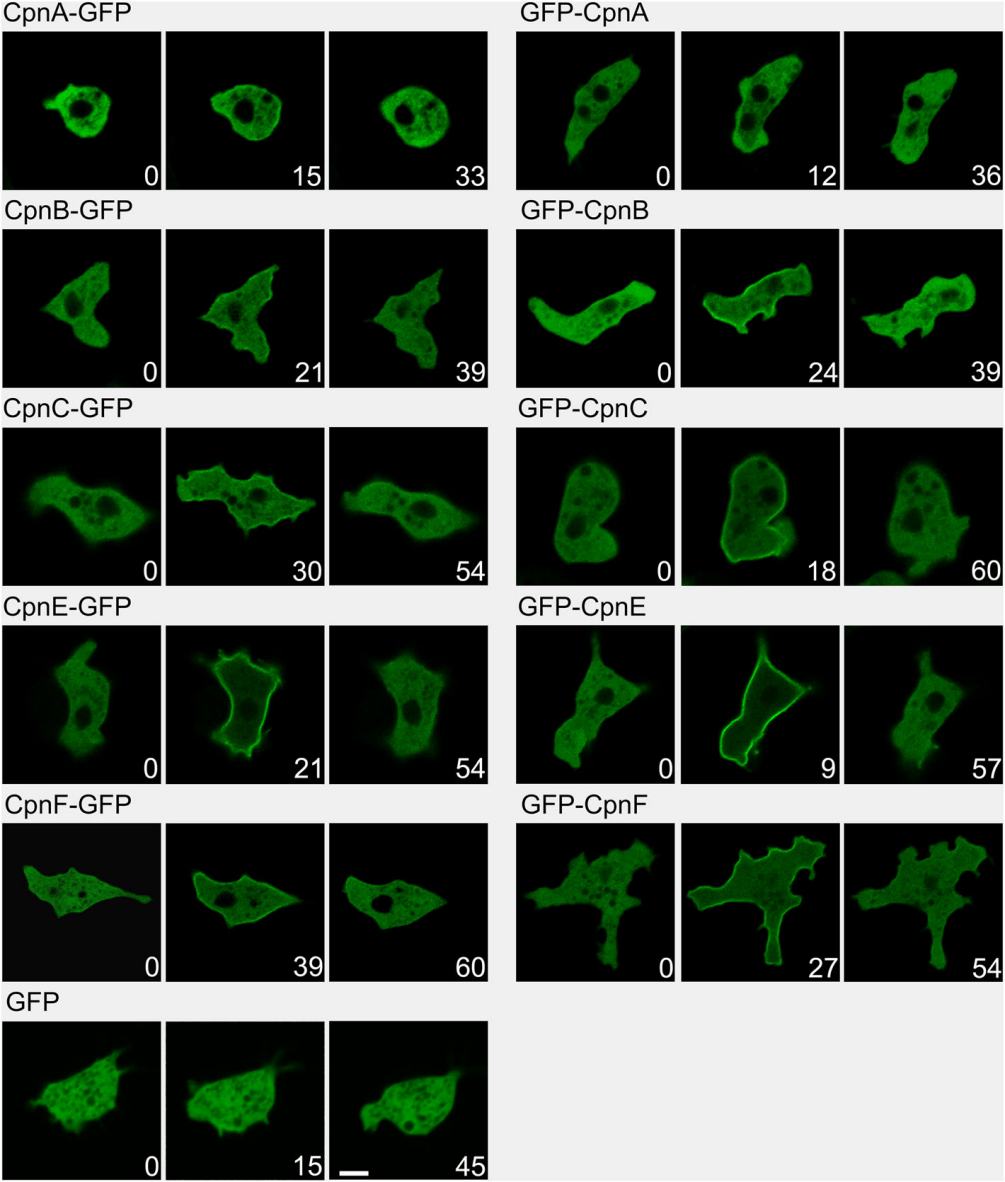
GFP-tagged copines translocate to the plasma membrane in response to cAMP stimulation. Cells expressing GFP or a GFP-tagged copine were pulsed with 50 nM cAMP every 6 min for 5 h prior to plating cells in glass bottom dishes with 2 mM caffeine and 2 mM CaCl_2_. Cells were stimulated with cAMP and time-lapse images were obtained every 3 s after stimulation, time (seconds) of the image is noted in bottom right corner of each micrograph. Images were obtained with a Nikon Eclipse Ti inverted laser scanning confocal microscope using a 60× oil immersion objective. Representative images are shown. Scale bar = 5 μm. Time-lapse images were converted to movies at 2 frames per second using ImageJ (see Additional files 2, 3, 4, 5, 6, 7, 8, 9, 10, 11 and 12)


Once we observed translocation of the copines in response to calcium ionophore treatment, we wanted to know if translocation could occur in response to a natural stimulus. Therefore, we took advantage of the *Dictyostelium* life cycle. In the absence of nutrients, unicellular amoeba will secrete and respond to cAMP. Cells will secrete cAMP approximately every 6 min, which in turn results in the cells aligning head-to-tail and streaming into mounds, with mound formation occurring approximately 6–8 h later. In response to cAMP pulses, intracellular calcium levels increase and reach a peak concentration at approximately 10–15 s after each pulse [11]. This developmental process can be mimicked in the laboratory: cells were starved for 1 h and pulsed with 50 nM cAMP every 6 min for 5 h in shaking suspension. Cells were then plated on glass bottom dishes in buffer with 2 mM caffeine and 2 mM CaCl_2_. Cells are unable to synthesize cAMP in the presence of caffeine; therefore, the cells are unable to signal each other in the presence of caffeine. Cells were globally stimulated with cAMP and then imaged every 3 s after stimulation. All the GFP-tagged copines were found in the cytoplasm prior to stimulation and then translocated to the plasma membrane in response to cAMP stimulation (Fig. 3 and Additional files 2, 3, 4, 5, 6, 7, 8, 9, 10, 11 and 12). The terminus at which the GFP was located did not appear to affect the timing or magnitude of the translocation of the copine.

**Additional file 2:** GFP-CpnA translocates to the plasma membrane in response to cAMP. Cells were stimulated with cAMP and time-lapse images were obtained every 3 s after stimulation. Time-lapse images were converted to avi format at 2 frames per second using ImageJ. (AVI 49 kb)



**Additional file 3:** CpnA-GFP translocates to the plasma membrane in response to cAMP. Cells were stimulated with cAMP and time-lapse images were obtained every 3 s after stimulation. Time-lapse images were converted to avi format at 2 frames per second using ImageJ. (AVI 44 kb)



**Additional file 4:** GFP- CpnB translocates to the plasma membrane in response to cAMP. Cells were stimulated with cAMP and time-lapse images were obtained every 3 s after stimulation. I Time-lapse images were converted to avi format at 2 frames per second using ImageJ. (AVI 39 kb)



**Additional file 5:** CpnB-GFP translocates to the plasma membrane in response to cAMP. Cells were stimulated with cAMP and time-lapse images were obtained every 3 s after stimulation. Time-lapse images were converted to avi format at 2 frames per second using ImageJ. (AVI 43 kb)



**Additional file 6:** GFP-CpnC translocates to the plasma membrane in response to cAMP. Cells were stimulated with cAMP and time-lapse images were obtained every 3 s after stimulation. Time-lapse images were converted to avi format at 2 frames per second using ImageJ. (AVI 49 kb)



**Additional file 7:** CpnC-GFP translocates to the plasma membrane in response to cAMP. Cells were stimulated with cAMP and time-lapse images were obtained every 3 s after stimulation. Time-lapse images were converted to avi format at 2 frames per second using ImageJ. (AVI 52 kb)



**Additional file 8:** GFP-CpnE translocates to the plasma membrane in response to cAMP. Cells were stimulated with cAMP and time-lapse images were obtained every 3 s after stimulation. Time-lapse images were converted to avi format at 2 frames per second using ImageJ. (AVI 59 kb)



**Additional file 9:** CpnE-GFP translocates to the plasma membrane in response to cAMP. Cells were stimulated with cAMP and time-lapse images were obtained every 3 s after stimulation. Time-lapse images were converted to avi format at 2 frames per second using ImageJ. (AVI 58 kb)



**Additional file 10:** GFP-CpnF translocates to the plasma membrane in response to cAMP. Cells were stimulated with cAMP and time-lapse images were obtained every 3 s after stimulation. Time-lapse images were converted to avi format at 2 frames per second using ImageJ. (AVI 79 kb)



**Additional file 11:** CpnF-GFP translocates to the plasma membrane in response to cAMP. Cells were stimulated with cAMP and time-lapse images were obtained every 3 s after stimulation. Time-lapse images were converted to avi format at 2 frames per second using ImageJ. (AVI 58 kb)



**Additional file 12:** GFP does not translocate to the plasma membrane in response to cAMP. Cells were stimulated with cAMP and time-lapse images were obtained every 3 s after stimulation. Time-lapse images were converted to avi format at 2 frames per second using ImageJ. (AVI 38 kb)


**Fig. 4:**
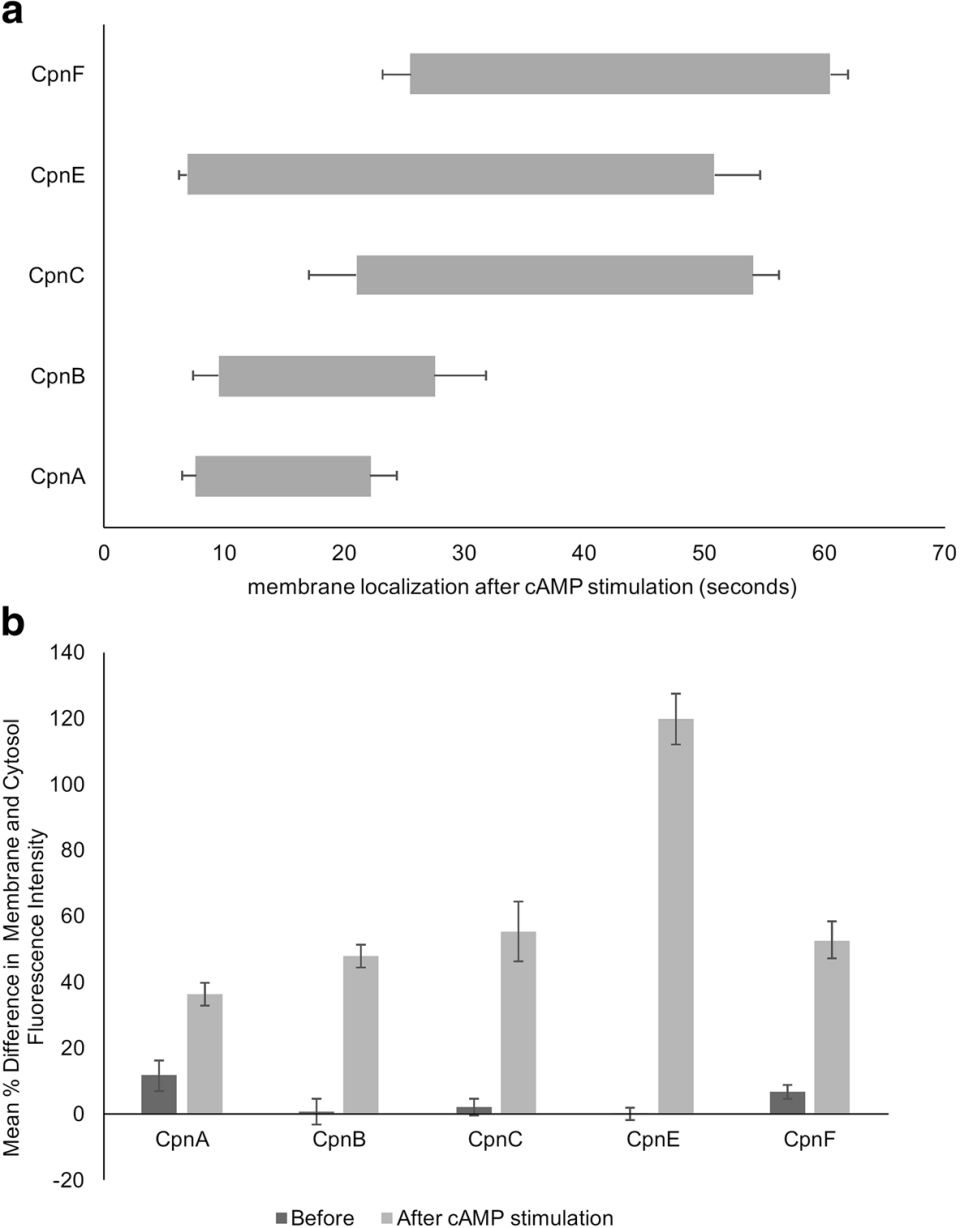
Copines translocate to the plasma membrane with various on and off times and magnitudes after cAMP stimulation. **a** Time-lapse images of individual cells were obtained as described in Fig. 3 and were analyzed by determining the time at which the GFP-tagged copine was first observed to be enriched on the plasma membrane and the time at which the GFP-tagged copine was no longer enriched on the plasma membrane. Times for at least six and up to 21 cells for each copine regardless of the location of the GFP were averaged. Error bars = standard error. **b** Time-lapse images of individual cells were analyzed for mean fluorescence intensity in the cytosol and on the plasma membrane. The percent difference between the mean fluorescence intensity on the plasma membrane and the cytosol was calculated using images taken before cAMP stimulation and images in which the most membrane localization was observed after cAMP stimulation. The percent differences were averaged for six cells for each copine. Error bars = standard error.

The time it took for translocation to start after cAMP stimulation varied among the different copines (Fig. 4a). CpnA, CpnB, and CpnE responded fast with membrane localization observed at an average of ~ 7–9 s after stimulation, while CpnC and CpnF membrane localization occurred later with an average of ~ 21 and 25 s, respectively. The time spent on the membrane also varied among the different copines (Fig. 4a). CpnA and CpnB stayed on the membrane for an average of 15 and 18 s, respectively, while CpnC and CpnF stayed on the membrane longer (~ 33 and 35 s). CpnE stayed on the membrane the longest with an average of 44 s (Fig. 4a). In addition to differences in the timing of membrane translocation in response to cAMP, the magnitude of the response differed among the copines. CpnA’s response to cAMP stimulation was extremely subtle, in that it was difficult to see membrane localization in the images captured and more often than not, no membrane localization was observed in response to cAMP. In contrast, CpnE’s response to cAMP was dramatic and easily captured (Fig. 3). To quantify these differences, we measured the fluorescence intensity in the cytosol and on the plasma membrane for individual cells before and after cAMP stimulation. We then used these measurements to calculate the percent difference in fluorescence intensity between the plasma membrane and cytosol before and after cAMP stimulation for each copine (Fig. 4b). CpnE showed the largest percent difference in fluorescence intensity (119.7% ± 5.4) between the plasma membrane and cytosol after cAMP stimulation, indicating that much of the protein translocated from the cytosol to the plasma membrane. CpnA showed the smallest percent difference (36.1% ± 3.4). These differences in the timing (Fig. 4a) and magnitude of the membrane localization (Fig. 4b) in response to cAMP among the copines suggests that the copines have varying calcium sensitivities and/or lipid-binding activities.

### Copines exhibit both calcium-dependent and calcium-independent binding to membranes

**Fig. 5:**
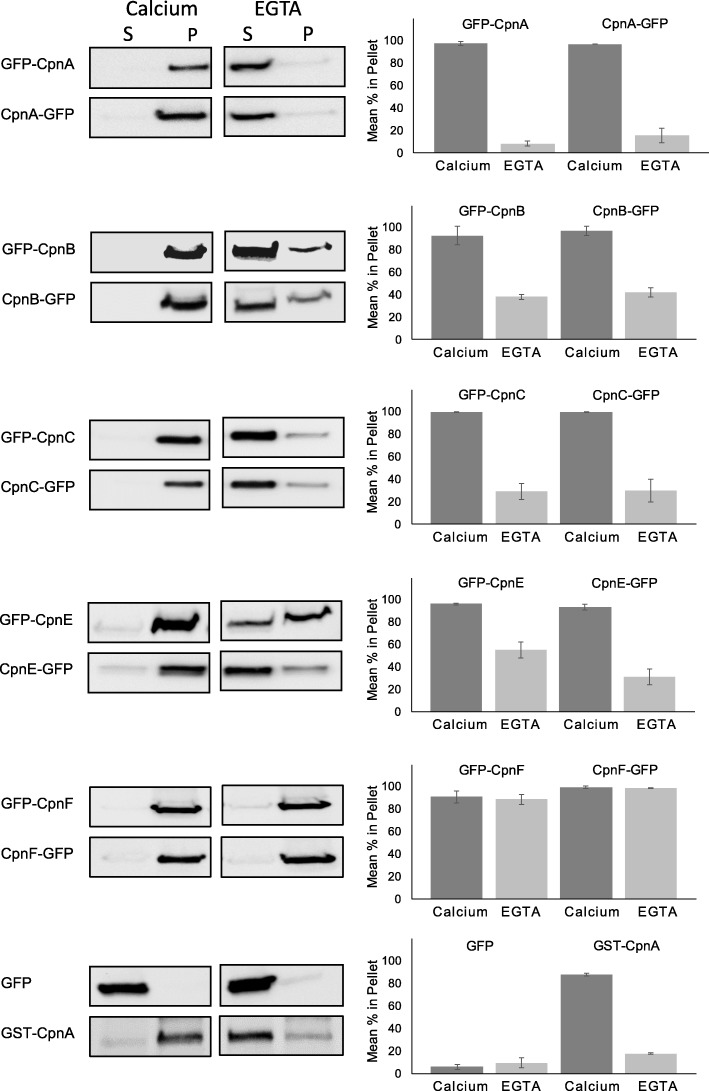
Copines have different calcium-dependent and -independent membrane-binding properties. Cells expressing GFP, a GFP-tagged copine, or GST-CpnA were disrupted and all membranes were pelleted by ultracentrifugation in the presence (calcium) or absence of calcium (EGTA). The supernatant samples (S) and pelleted membranes samples (P) were analyzed by Western blot with an antibody to GFP. Three blots were analyzed by densitometry and the average percentage of the protein found in the pellet was graphed. Error bars = standard error

Because each of the copine proteins responded to cAMP stimulation with distinct timing, we performed in vitro membrane-binding assays to investigate their calcium-dependent membrane-binding properties. In our previous work, we showed that endogenous CpnA and overexpressed GFP-CpnA bind to disrupted cell membranes in a calcium-dependent manner [2]. In this study, we used a similar assay to investigate the calcium-dependent membrane-binding properties of GFP-tagged versions of CpnA, CpnB, CpnC, CpnE, and CpnF. First, cells were disrupted and then treated with either calcium or EGTA, and then all membranes were pelleted by ultracentrifugation. The pellets and supernatants were analyzed by Western blot analysis using an antibody to GFP. These experiments were repeated at least three times and the Western blots were analyzed by densitometry. In the presence of calcium, GFP-tagged versions of CpnA, CpnB, CpnC, CpnE, and CpnF were mostly found associated with the pelleted membranes (90.4% ± 5.3–99.1% ± 0.46) and nearly completely absent from the supernatants (Fig. 5). In the presence of EGTA, a small amount of GFP-tagged CpnA (8.2% ± 2.0, 15.6% ± 6.6) was found in the pellet. These results indicate that CpnA exhibits mostly calcium-dependent membrane-binding. CpnB, CpnC, and CpnE exhibited both calcium-dependent and -independent membrane-binding with the amount of protein found in the pellet ranging from 28.9% ± 7.3–54.7% ± 6.9 in the presence of EGTA. In contrast, almost all of the GFP-tagged CpnF was found in the pellet (88.2% ± 4.3, 98.1% ± 0.57) in the presence of EGTA (Fig. 5), indicating CpnF exhibits mostly calcium-independent membrane-binding. The terminus at which the GFP was located did not appear to affect the copines’ membrane-binding properties (Fig. 5). We also performed this membrane-binding assay with cells expressing GFP and found that in the presence of calcium or EGTA, GFP was nearly absent from the membrane pellet and mostly in the supernatant. Furthermore, cells expressing GST-tagged CpnA were also examined and GST-CpnA exhibited similar binding properties to the GFP-tagged CpnA (Fig. 5). In this membrane-binding assay, we are pelleting disrupted cells and therefore in addition to all membranes, organelles that have not been disrupted and cytoskeletal elements are also being pelleted. Therefore, some of the copine protein found in the pellet may be in the nucleus and/or associated with the cytoskeleton.

**Fig. 6:**
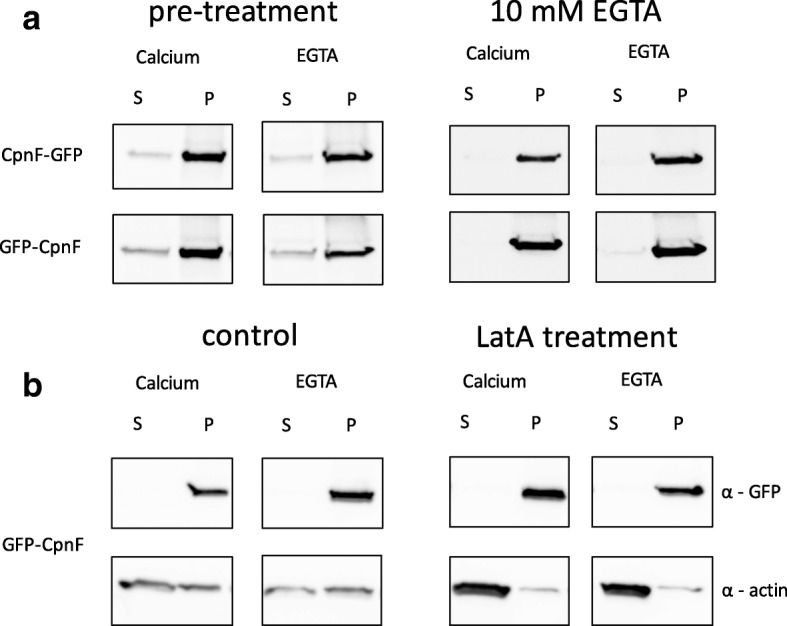
Pre-treatment, higher concentration of EGTA, and actin depolymerization does not change CpnF binding to membranes. **a** Cells expressing GFP-CpnF and CpnF-GFP were either disrupted in the presence of EGTA and calcium (pre-treatment) or disrupted and then treated with EGTA (10 mM) and calcium (2 mM). All membranes were pelleted by ultracentrifugation. The supernatant samples (S) and pelleted membranes samples (P) were analyzed by Western blot with an antibody to GFP. **b** Cells expressing GFP-CpnF were treated with Latrunculin A (LatA) or DMSO (control) for 30 min prior to disruption. All membranes were pelleted by ultracentrifugation. The supernatant samples (S) and pelleted membranes samples (P) were analyzed by Western blot with an antibody to GFP (α-GFP) and an antibody to actin (α-actin)

The results of the in vitro binding assay for CpnF were unexpected given that we observed the translocation of CpnF from the cytosol to the plasma membrane in response to cAMP stimulation. Calcium ionophore treatment also resulted in membrane binding, although it was difficult to trigger, requiring higher concentrations of calcium and more time. One explanation for the native membrane binding result is that once CpnF binds to membranes in response to calcium, its binding becomes calcium-independent. To test this idea, we repeated the native membrane binding assays with cells expressing GFP-CpnF and CpnF-GFP using a slightly different protocol. This time we added the EGTA and calcium to the buffer before the disruption process, presumably preventing the binding to membranes in the first place. We also repeated the experiment again, but with a higher concentration of EGTA (from 5 mM to 10 mM). In both cases, the GFP-CpnF and CpnF-GFP still pelleted with membranes (Fig. 6a). In addition, to determine if CpnF was pelleting with the actin cytoskeleton, we performed these assays again; however, prior to cell disruption, we treated the cells expressing GFP-CpnF with Latrunculin A (LatA) to depolymerize actin filaments. Treatment with LatA resulted in less actin in the pellet, but did not change the amount of CpnF found in the pellet in the presence of calcium or EGTA (Fig. 6b), indicating that CpnF was not pelleting with actin filaments.

### Copines bind to a variety of acidic phospholipids

**Fig. 7:**
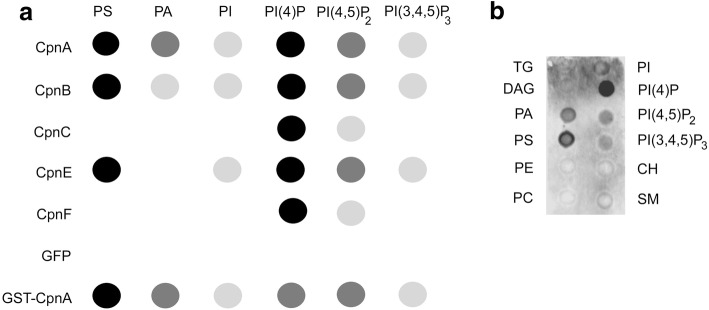
Copines vary in their binding to specific acidic phospholipids. GFP-tagged copines were isolated by immunoprecipitation and the protein/antibody complex was incubated with commercially available lipid dot blots in the presence of calcium. Binding was detected with a secondary antibody conjugated to HRP and chemiluminescence. **a** Multiple blots for each copine protein were analyzed and the combined data is represented as grayscale dots, with white referring to no signal observed and black referring to the most intense signal. **b** An image of a single dot blot showing relevant lipids bound by GFP-CpnA. Lipid abbreviations: phosphatidylserine (PS), phosphatidic acid (PA), phosphatidylinositol (PI), phosphatidylinositol 4-phosphate (PI(4)P), phosphatidylinositol 4, 5-phosphate (PI(4,5)P_2_) and phosphatidylinositol 3, 4,5-phosphate (PI(3,4,5)P_3_), triglyceride (TG), diacylglycerol (DAG), phosphatidylethanolamine (PE), phosphatidylcholine (PC), cholesterol (CH), and sphingomyelin (SM)

To identify the specific phospholipids copines bind, we performed protein-lipid overlay assays with Echelon membrane lipid strips that contain biologically significant lipid dots. GFP-tagged versions of copines were purified by immunoprecipitation with an antibody to GFP and incubated with the Echelon membrane lipid strips in the presence of calcium to determine protein-lipid interactions. Copines, along with the antibody to GFP, bound to phospholipids on the lipid dot blot were detected by an HRP-conjugated anti-rabbit antibody and chemiluminescence. Representative blots for each of the copines are shown in Additional file 13. Dot blot assays were performed three or more times for each of the GFP-tagged copines and each dot on a blot was given a grayscale rating from white to black, with white referring to no signal observed and black referring to the most intense signal on the blot. Although we saw some variation among these dot ratings on the blots with the same copine protein, the location of the GFP tag did not appear to influence the specific lipids bound by the copine. Therefore, we combined the ratings from the blots with the same copine protein, regardless of the location of the GFP tag, and summarized the data in Fig. 7. Only phospholipids that showed consistent copine binding on multiple blots were included. CpnA, CpnB, and CpnE were found associated with a variety of acidic phospholipids, with the strongest binding to phosphatidylserine (PS) and phosphatidylinositol 4-phosphate (PI(4)P) and with less binding to phosphatidylinositol (PI), other phosphorylated versions of PI, PI(4,5)P_2_ and PI(3,4,5)P_3_, and phosphatidic acid (Fig. 7). CpnC and CpnF exhibited no binding to PS, strong binding to PI(4)P, and weak binding to PI(4,5)P_2_. None of the copines bound to triglyceride, diacylglycerol, phosphatidylcholine, phosphatidylethanolamine, cholesterol, and sphingomyelin. We also used purified GST-CpnA as a control and found that GST-CpnA behaved similarly to the immunoprecipitated GFP-tagged CpnA, binding to PS and PIP strongly. GFP immunoprecipitated from cells expressing GFP did not bind strongly to any of the lipids on the blot (Fig. 7). Some of the copines also bound to phosphatidylglycerol, cardiolipin, and sulfatide (Additional file 13), but the binding was not consistent across multiple blots. Furthermore, GFP alone was sometimes observed to bind to sulfatide.

## Discussion

Copines are found in a wide variety of eukaryotic organisms and are characterized by two C2 domains followed by an A domain. Because of their domain structure, copines have been hypothesized to be involved in signaling pathways by binding to membranes in response to increases in calcium through their C2 domains and bringing target proteins to membranes through interactions with their A domains [10]. Therefore, different copines could have roles in different signaling pathways dependent on their calcium sensitivities, lipid specificities, and target proteins. Multiple copine genes exist in various organisms ranging from two in *Paramecium* to nine in humans. Among genetically tractable model organisms, *Dictyostelium* provides an ideal model organism to study copines because *Dictyostelium* has six different copine genes, while *Arabidopsis* has three, *C.elegans* has two, and yeast and *Drosophila* have none. Using *Dictyostelium* to study copine function also allows for the study of copines in a single-celled and multicellular organism.

Previously, we focused our studies on one of the copine proteins in *Dictyostelium*, CpnA [2, 12–14]. In the present study, we have expanded our investigations to include four additional copines. We expressed all of the copines with a GFP tag in *Dictyostelium* and found that CpnB, CpnC, CpnE, and CpnF have similar localization patterns to CpnA in that they are cytoplasmic in live cells and associated with membranes in fixed cells. The only major difference we observed was that CpnB, CpnC, and CpnF were also found in the nucleus.

The major new finding with regard to localization was that all five copines translocate from the cytosol to the plasma membrane and back to the cytosol in response to cAMP, the extracellular signaling molecule used to signal cells to aggregate during development. Because cAMP stimulation leads to a transient increase in calcium concentration, this result was not unexpected. However, the timing and magnitude of the translocation in response to cAMP was quite different among the five copines, suggesting that each copine responds differently to the same signal and therefore has a distinct function. Mammalian copines are also capable of translocation from the cytosol to the plasma membrane in response to a rise in intracellular calcium concentration [4]. Like the *Dictyostelium* copines, the mammalian copines studied showed differences in rates of translocation in response to ionomycin treatment. In addition, only a subset of the mammalian copines tested translocated to membranes in response to methacholine stimulation in human embryonic kidney-293 cells [4], indicating mammalian copines respond differently to distinct signals. Reinhard et al. [6] reported that the mammalian Copine-6, exclusively expressed in brain, translocates from the cytosol to dendritic spines in mouse hippocampal neurons in response to synaptic activity and mediates changes in dendritic spine morphology important for learning and memory.

Consistent with the differences we observed in the responses of the copines to cAMP stimulation, we also found differences in their membrane binding properties with respect to calcium sensitivities and lipid specificities. To highlight these different properties among the copines, we have summarized our data in Table 1. None of the copines exhibited the exact same properties as another copine, so each copine was unique. However, CpnC and CpnF were the most similar, sharing all properties outlined in Table 1, except for one; CpnC displayed some calcium-dependent membrane-binding in our in vitro membrane binding assays, while CpnF displayed almost none, making it different from all of the copines. The only properties that were correlated with each other for all five copines were membrane translocation on times and lipid specificities. CpnA, CpnB, and CpnE translocated to the plasma membrane quickly in response to cAMP and bound strongly to PS and PIP, while CpnC and the CpnF took longer to translocate after cAMP stimulation and bound strongly to PIP, but not PS. This suggests that perhaps the differences in the timing of translocation to the membrane of copines in response to cAMP may have more to do with their lipid specificities than calcium sensitivities. In fact, our results suggest that CpnC and CpnF translocate to the plasma membrane after the intracellular calcium level has peaked. Reports of the time at which intracellular calcium levels peak after cAMP stimulation in *Dictyostelium* range from 6.5 to 15 s, while reports of the time at which intracellular calcium levels return to normal range from 19 to 25 s [25–28].

**Table 1:** Summary of membrane binding properties of *Dictyostelium* copines

	Nucleus	On Membrane	Off Membrane	Magnitude	Lipids	Ca^2+^- dep.
CpnA	no	fast	fast	low	PS, PIP	high
CpnB	yes	fast	fast	medium	PS, PIP	medium
CpnC	yes	slow	slow	medium	PIP	medium
CpnE	no	fast	slow	high	PS, PIP	medium
CpnF	yes	slow	slow	medium	PIP	low

C2 domains consist of approximately 130 residues and are comprised of an eight-stranded antiparallel β-sandwich consisting of two four-stranded β-sheets. Variations in the amino acids within the interconnecting surface loops have been found to confer different calcium and lipid-binding properties [7]. Furthermore, not all C2 domains have retained the ability to bind calcium, making some C2 domains calcium-independent lipid-binding domains. Some multiple C2 domain proteins have been shown to have one calcium-dependent and one calcium-independent C2 domain, and therefore, display both calcium-dependent and calcium-independent lipid-binding [29]. Studies with mammalian copines indicate that only one of the C2 domains is responsible for membrane localization [30]. To determine if differences in C2 domain sequences among the copines could be correlated with or could predict localization and/or lipid-binding properties, we determined the percent amino acid identity among the C2 domains of the *Dictyostelium* copines. CpnA and CpnF had the lowest (39%) and CpnB and CpnE had the highest (62%) percent amino acid identity. Our data also indicates that CpnA and CpnF are the least similar in that they do not share any of the localization or lipid-binding properties studied; however, other correlations between percent amino acid identity and the membrane-binding properties studied are not readily apparent (Additional file 14).

**Fig. 8:**
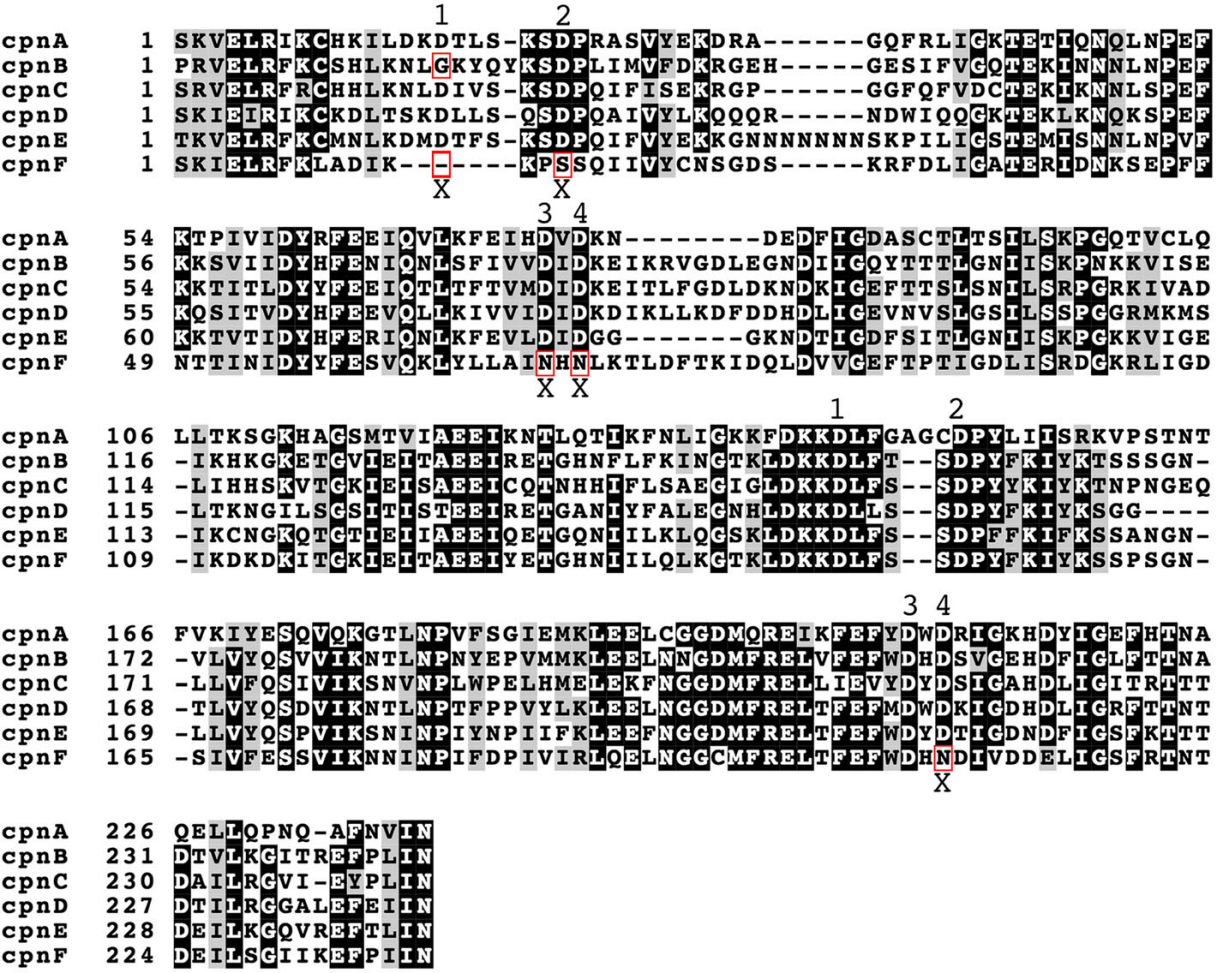
The C2 domains of CpnF are missing key aspartates. The amino acids that make up the two C2 domains of the six *Dictyostelium* copines were identified using the NCBI Conserved Domain Search. The two C2 domains and intervening sequences for all the copines were aligned using Clustal Omega. BoxShade was used to indicate conserved residues within the aligned sequences. Black boxes indicate where at least five out of the six copines have the identical amino acid, while gray boxes indicate where at least five out of the six copines have a similar amino acid. Four key aspartates thought to be involved in calcium binding in each C2 domain are indicated with a number. Locations were these aspartates are missing are indicated with an X and highlighted with a red box

Analysis of the amino acid sequences of the C2 domains of the *Dictyostelium* copines (Fig. 8) reveals that the *Dictyostelium* copines appear to have the eight aspartate residues thought to be involved in calcium ion binding [30]. The only exceptions were found in CpnB and CpnF, with CpnB missing the first aspartate in the first C2 domain and CpnF missing all four aspartates in the first C2 domain and one in the second C2 domain (Fig. 8). Three of the missing aspartates in CpnF are replaced with the positively charged amino acid, arginine. These differences in the CpnF sequence from other copine sequences may account for the lack of calcium-dependent binding to disrupted cell membranes we observed with CpnF, but does not explain why we observed translocation of CpnF from the cytosol to plasma membrane in calcium ionophore treated and cAMP stimulated cells. Other regions of the protein may play a part in membrane localization; some of the mammalian copines have been shown to require the region between the C2 domains and the A domain for membrane localization [30]. In addition, translocation could be triggered by a modification of CpnF itself or a change in the lipid or protein composition of the membrane in response to these stimuli.

All of the copines bind some form of phosphorylated phosphatidylinositols, which are known regulators of the actin cytoskeleton and membrane trafficking [31–33]. In response to cAMP signaling in *Dictyostelium*, phosphatidylinositol 3-kinase translocates to the leading edge of the plasma membrane and phosphorylates PI(4,5)P_2_ to PI(3,4,5)P_3_, which interacts with a number of downstream targets that regulate actin cytoskeleton dynamics in chemotaxis [31]. However, the complete signaling pathway from cAMP to changes in the actin cytoskeleton is not fully characterized. We have recent data that indicates that CpnA binds directly to actin filaments in a calcium-dependent manner and *cpnA*^*−*^ cells have severe defects in cAMP chemotaxis suggesting that CpnA may be part of the missing link between cAMP and actin filament reorganization. In the future, we will use these GFP-tagged copines to identify their protein binding partners. We also plan to create single and multiple copine gene knockout mutants in *Dictyostelium* to explore the role of copines in chemotaxis and to identify other unique and overlapping functions.

## Conclusions

Copines are ubiquitous proteins found in most eukaryotic organisms. However, their exact function is unknown. Copines are soluble calcium-dependent membrane-binding proteins and are hypothesized to translocate to membranes in response to signaling pathways that trigger a rise in calcium concentration; yet, few in vivo studies have demonstrated this translocation. Here, we show that all five of the *Dictyostelium* copines studied translocated from the cytosol to the plasma membrane and back in response to stimulation by the extracellular chemotaxis signaling molecule, cAMP, but with varying magnitudes and on and off times. Each copine also exhibited unique membrane-binding properties with distinct calcium-sensitivity and lipid-specificity. These data suggest that each copine plays a different role in various processes regulated by calcium and phospholipid signaling.

## Additional files

Additional file 1: GFP-tagged copine expression in *Dictyostelium* verified by Western blot. Cells expressing GFP or a GFP-tagged copine (2 × 10^6^ cells) were analyzed by Western blot using an antibody to GFP. (PDF 135 kb)

Additional file 13: GFP-tagged copines bind to a variety of acidic phospholipids. GFP-tagged copines were isolated by immunoprecipitation and the protein/antibody complex was incubated with commercially available lipid dot blots in the presence of calcium. Binding was detected with an antibody conjugated to HRP and chemiluminescence. A representative dot blot is shown for each GFP-tagged protein and GST-CpnA. Lipid abbreviations: phosphatidylserine (PS), phosphatidic acid (PA), phosphatidylinositol (PI), phosphatidylinositol 4-phosphate (PI(4)P), phosphatidylinositol 4, 5-phosphate (PI(4,5)P_2_) and phosphatidylinositol 3, 4,5-phosphate (PI(3,4,5)P_3_), triglyceride (TG), diacylglycerol (DAG), phosphatidylethanolamine (PE), phosphatidylcholine (PC), cholesterol (CH), and sphingomyelin (SM), phosphatidylglycerol (PG), cardiolipin (CL), and sulfatide (SF). (PDF 305 kb)

Additional file 14: Percent of C2 domain amino acid identity. The two C2 domains and intervening sequences for all the *Dictyostelium* copines were aligned and the percent amino acid identity calculated using Clustal Omega. (DOCX 13 kb)

## Abbreviations

Cpn: Copine
PI(4)P: Phosphatidylinositol 4-phosphate
PS: Phosphatidylserine
VWA: Von Willebrand A
PI(3, 4, 5)P_3_: Phosphatidylinositol 3, 4, 5-trisphosphate
PI(4, 5)P_2_: Phosphatidylinositol 4, 5-bisphosphate
