# Supplementary figures and images for: Cyclic AMP signaling in Dictyostelium promotes the translocation of the copine family of calcium-binding proteins to the plasma membrane
Source: BMC Cell Biol. 2018 Jul 16;19:13. doi: 10.1186/s12860-018-0160-5 (PMC6048903; doi:10.1186/s12860-018-0160-5)

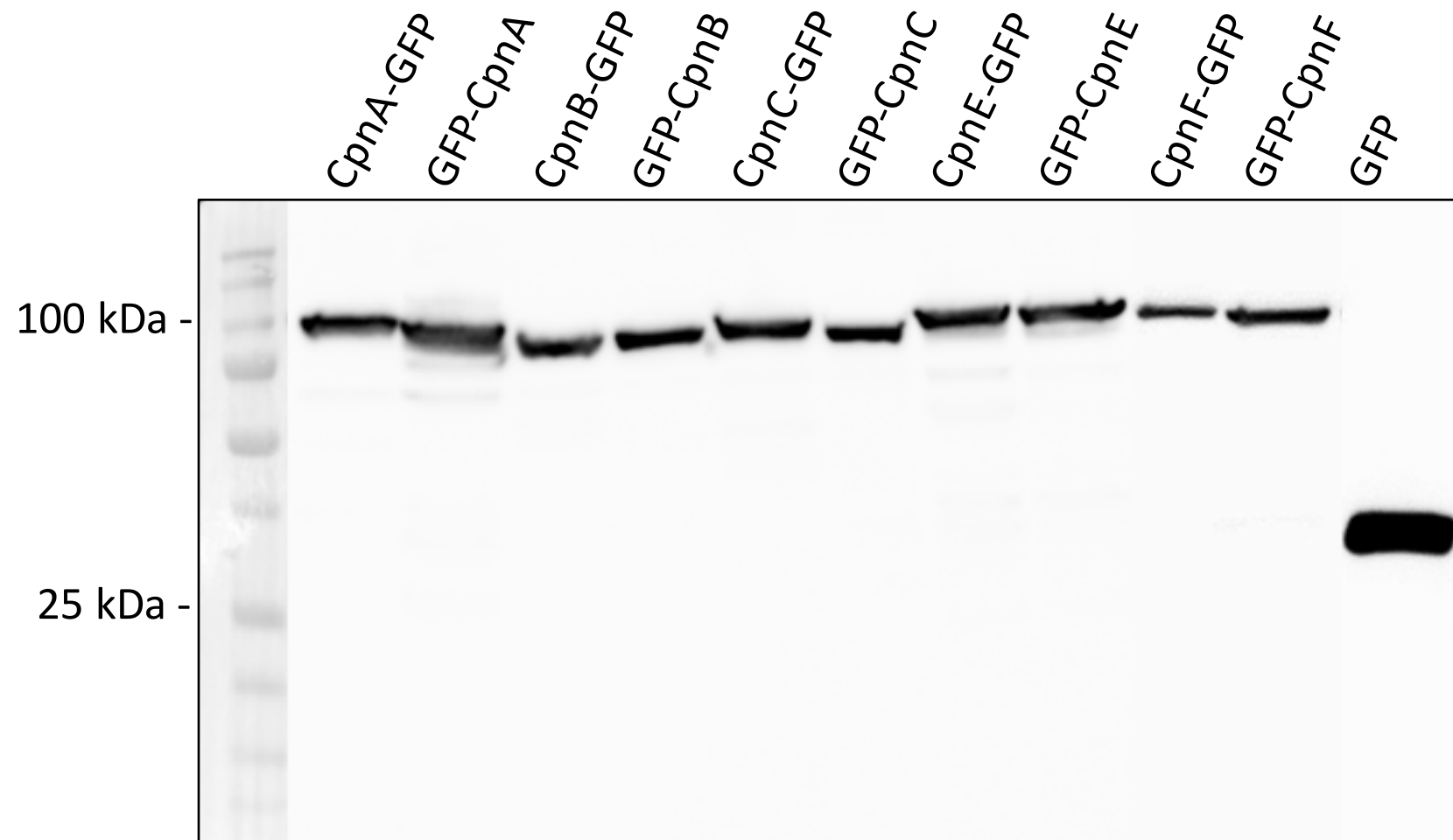

Supplement: Supplementary file 12 — GFP-tagged copine expression in Dictyostelium verified by Western blot. Cells expressing GFP or a GFP-tagged copine (2 × 10^6^ cells) were analyzed by Western blot using an antibody to GFP. (PDF 135 kb) [file 12860_2018_160_MOESM1_ESM.pdf]

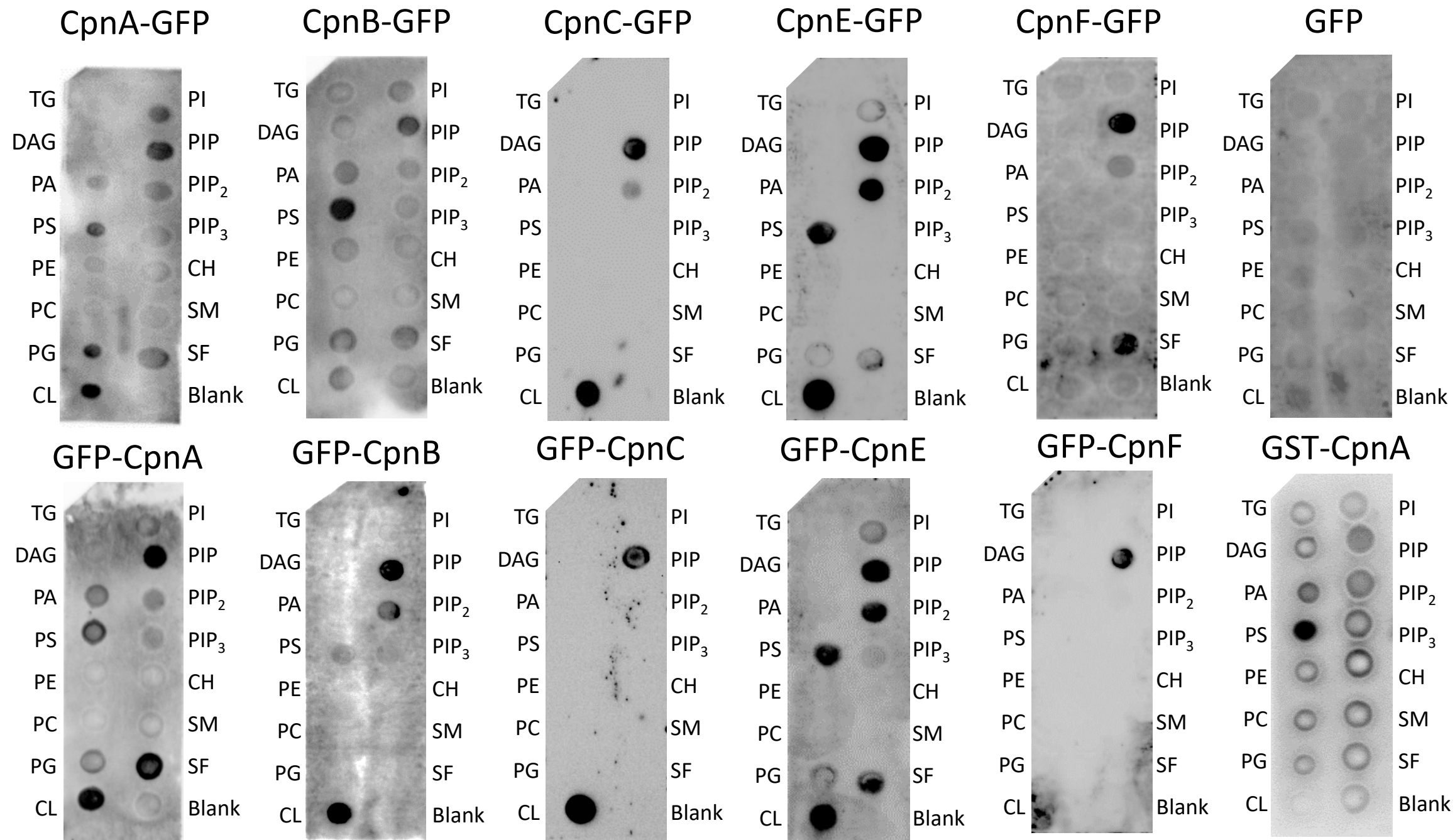

Supplement: Supplementary file 13 — GFP-tagged copines bind to a variety of acidic phospholipids. GFP-tagged copines were isolated by immunoprecipitation and the protein/antibody complex was incubated with commercially available lipid dot blots in the presence of calcium. Binding was detected with an antibody conjugated to HRP and chemiluminescence. A representative dot blot is shown for each GFP-tagged protein and GST-CpnA. Lipid abbreviations: phosphatidylserine (PS), phosphatidic acid (PA), phosphatidylinositol (PI), phosphatidylinositol 4-phosphate (PI(4)P), phosphatidylinositol 4, 5-phosphate (PI(4,5)P2) and phosphatidylinositol 3, 4,5-phosphate (PI(3,4,5)P3), triglyceride (TG), diacylglycerol (DAG), phosphatidylethanolamine (PE), phosphatidylcholine (PC), cholesterol (CH), and sphingomyelin (SM), phosphatidylglycerol (PG), cardiolipin (CL), and sulfatide (SF). (PDF 305 kb) [file 12860_2018_160_MOESM13_ESM.pdf]
