# Supplementary material for: Cyclic AMP signaling in Dictyostelium promotes the translocation of the copine family of calcium-binding proteins to the plasma membrane
Source: BMC Cell Biol. 2018 Jul 16;19:13. doi: 10.1186/s12860-018-0160-5 (PMC6048903; doi:10.1186/s12860-018-0160-5)
Supplement: Supplementary file 14 — Percent of C2 domain amino acid identity. The two C2 domains and intervening sequences for all the Dictyostelium copines were aligned and the percent amino acid identity calculated using Clustal Omega. (DOCX 13 kb) [file 12860_2018_160_MOESM14_ESM.docx]

Additional file 14. Percent of C2 domain amino acid identity

|  | CpnA | CpnB | CpnC | CpnD | CpnE | CpnF |
| --- | --- | --- | --- | --- | --- | --- |
| CpnA | 100 | 46 | 41 | 49 | 49 | 39 |
| CpnB | 46 | 100 | 55 | 51 | 62 | 48 |
| CpnC | 41 | 55 | 100 | 50 | 56 | 45 |
| CpnD | 49 | 51 | 50 | 100 | 52 | 46 |
| CpnE | 49 | 62 | 56 | 52 | 100 | 55 |
| CpnF | 39 | 48 | 45 | 46 | 55 | 100 |
